# Supplementary material for: Optimizing costs and sustainability for gamma knife radiosurgery: A cost and breakeven analysis at India’s largest neurosurgery centre
Source: PLoS One. 2025 Jan 13;20(1):e0306159. doi: 10.1371/journal.pone.0306159 (PMC11729947; doi:10.1371/journal.pone.0306159)
Supplement: Attachment — Submitted filename: Response to Reviewers.docx [file pone.0306159.s001.docx]

| **S.No.** | **Reviewer** | **Observations/Comments** | **Response** | **Line No.** |
| --- | --- | --- | --- | --- |
| 1. |  | Please ensure that your manuscript meets PLOS ONE's style requirements, including those for file naming. | Rechecked and it is found to be meeting PLOS One style requirements. | NA |
| 2 |  | We note that your Data Availability Statement is currently as follows:  All relevant data are within the manuscript and its Supporting Information files. | The relevant dataset has been included and attached in Supporting information file. | NA |
| 3 |  | Please ensure that you refer to Figure 1 in your text as, if accepted, production will need this reference to link the reader to the figure. | Necessary changes done and linked in text. | 218 |
| 4 |  | Please review your reference list to ensure that it is complete and correct. | Reviewed and found to be correct & complete. | NA |
| B. **Additional Editor Comments:** | | | | |
| 1 |  | Your manuscript, "Optimizing costs and sustainability for Gamma Knife Radiosurgery: A cost and breakeven analysis at India's largest Neurosurgery Centre," addresses a critical and underexplored topic in healthcare economics. The study provides valuable insights into cost optimization and sustainability in the context of advanced neurosurgical care.  To enhance the manuscript further, consider elaborating on the methodology, particularly the classification of fixed and variable costs, to improve clarity and reproducibility. Expanding on the potential implications for patient access, clinical outcomes, and healthcare policy would strengthen the discussion. Additionally, comparisons with similar analyses from other regions or international contexts could provide a broader perspective and highlight the global relevance of your findings. Overall, your study is well-conceived and offers significant potential for guiding cost-effective practices in Gamma Knife Radiosurgery. | We have incorporated the detailed explanation in our methodology as well as in **Table 1** along with the reference to address your recommendations and enhance the methodological clarity.  Discussion has been also strengthened taking into consideration the inputs provided by the reviewers as well. However, additional similar analysis from the region and the globe could not be found during the review. | 109-122  365-372 |
| 1 | Reviewer #1 | Mention the software used for calculation. (I suppose all calculations were done in Microsoft Excel. Mention is along with the version of software) | Microsoft excel 2018 version | 107 |
| 2 |  | Nominal interest rate was not mentioned. Is it equivalent to the discount factor generally used in costing studies? | Nominal interest rate are the bank lending rates and was taken as 6% in this study, and thereafter real interest was calculated to be 2.9~3 %. In general, it is suggested that we use a real interest rate of 3%, as it has been found in a comprehensive set of cost effectiveness studies for the health sector (both developed and developing countries) and its use makes hospital costing consistent with the international literature.[8] | 139-143 |
| 3 |  | The author should provide a number of Human resources with their time contribution to GKRS. (May be in supplemental material) | Included in the supplementary data set file. |  |
| 4 |  | Is there any overhead charges for the administration department (which supports the overall organization where the facility is established) included in the cost analysis? If not please give reason (it may be included in limitation) | Administrative costs and other costs such as Land, Computer Facility, Medical Records, IT costs, etc. as depicted in Table 1 would have been insignificant cost heads in comparison to the overall cost and its identification would have taken significant resources, hence, excluded from the study. | 361-64 |
| 5 |  | Why 5 years cost was calculated? Where you have used it in analysis? | Five years cost was used for calculating breakeven analysis. This time frame was selected based on the projection that all the investments would be recouped within this period and thereafter, the facility is expected to start generating surplus considering that the life span of equipment is estimated to be 10 years. | 189-193 |
| 6 |  | In break-even analysis, the fixed cost = US$ 717,509 (INR5,05,12,665) (Capital Cost) is kept constant as numerator. Is it annual? Or per procedure? And in the denominator, procedure charge – variable cost (as per the equation on page 10, line 177) = 115000-96239 = 18761. So the breakeven point must be = 5,05,12,665/18761 = 2692, which is not equal to the break-even point mentioned in scenario 1 in Table 6. Can you explain how do you calculated it? | In break-even analysis, the fixed cost is kept constant as a numerator and is calculated as a capital cost per annum irrespective of number of procedures and the variable cost is per procedure.  There have been some errors with regards to the procedure cost in the table in scenario 1 & 2 and same has been corrected in the table. Approach demonstrated by you is the same that has been followed in the study. | Table 6 corrected  295 |
| 7 |  | I recommend that the author provide a raw data file with all the data required to reproduce the values mentioned in the manuscript as supplemental material. | As mentioned in supporting information file | NA |
| 8 |  | Label the X axis for figure 2. | Labelled as “Percentage of the total cost contributed by each cost centre” and updated in Figure 2 | NA |
| 9 |  | Figure legends were not given | Incorporated in Figure 2 | NA |
| 10 |  | In figure 3, please mention where actually the break-even point is (crossing the blue and red line, I suppose) | Figure 3 has been deleted as being redundant. The required information is provided in text and table itself, obviating the need for images. | NA |
| 11 |  | In Figure 3 C, the Y-axis shows one “0” extra. Is this a mistake, or did you get the results you expected? | Figure 3 has been deleted as being redundant. The required information is provided in text and table itself, obviating the need for images. | NA |
| 12 |  | The values mentioned in Figure 3 a,b titles are not in the table or text? Please explain who you derive this value. | Figure 3 has been deleted as being redundant. The required information is provided in text and table itself, obviating the need for images. | NA |
| 13 |  | Figure 3 quality is poor. (Maybe you can revise fonts and pixel size) | Figure 3 has been deleted as being redundant. The required information is provided in text and table itself, obviating the need for images. | NA |
|  | Reviewer #2: | I read with interest the study by Agrawal et al. The authors, in this study, analyze the costs related to the use of gamma knife radio surgery in the largest neurosurgery center of an Institute of National Importance, in Delhi, India. The reported data clearly reflect the legislative regulations adopted in India. The costs highlighted appear to be important, both those related to the operation of the equipment (building, water, refrigeration, energy) and those related to the dedicated personnel (healthcare personnel, technicians). The possible reduction of costs should be linked to a greater optimization of the use of the machine. Therefore, identifying patients with clear indications for this type of treatment, periodic maintenance checks and specifically trained personnel are actions that would lead to the optimization of the use of the treatment with possible reduction of costs. The comparison reported in the text between patients treated with GKRS and patients treated surgically does not seem pertinent to me. In patients undergoing surgical treatment there are numerous variants that must be considered (prolonged hospitalization, pharmacological therapy, complications). In my opinion this part could be removed from the text | Suggestion and comments have been included. | 365-372 |
