# Supplementary material for: Optimizing costs and sustainability for gamma knife radiosurgery: A cost and breakeven analysis at India’s largest neurosurgery centre
Source: PLoS One. 2025 Jan 13;20(1):e0306159. doi: 10.1371/journal.pone.0306159 (PMC11729947; doi:10.1371/journal.pone.0306159)
Supplement: Attachment — Submitted filename: Response to Reviewers - Revised.docx [file pone.0306159.s002.docx]

| **S.No.** |  | **Observations/Comments** | **Response** | **Line No.** |
| --- | --- | --- | --- | --- |
| 1. | Journal Requirements | Please review your reference list to ensure that it is complete and correct. If you have cited papers that have been retracted, please include the rationale for doing so in the manuscript text or remove these references and replace them with relevant current references. Any changes to the reference list should be mentioned in the rebuttal letter that accompanies your revised manuscript. If you need to cite a retracted article, indicate the article’s retracted status in the References list and also include a citation and full reference for the retraction notice. | List of references reviewed and has been amended with minor changes.  As on date, no retracted article found in the list. It was checked in PubMed and original publisher’s site.  No changes have been made to the list of references.  May please advise any specific change/s to be incorporated. | Included in list of references |
